# Decreased levels of the serum inflammatory biomarkers, sGP130, IL-6, sCRP and BAFF, are associated with increased likelihood of AIDS related Kaposi’s sarcoma in men who have sex with men

**DOI:** 10.17980/2018.45

**Published:** 2018

**Authors:** Rachel Bolanos, Otoniel Martinez-Maza, Zuo-Feng Zhang, Shehnaz Hussain, Mary Sehl, Janet S. Sinsheimer, Gypsyarn D’Souza, Frank Jenkins, Steven Wolinsky, Roger Detels

**Affiliations:** 1 Department of Epidemiology, Fielding School of Public Health, UCLA, Los Angeles, CA; 2 Departments of Obstetrics and Gynecology, and Microbiology, Immunology, and Molecular Genetics, David Geffen School of Medicine, UCLA, Los Angeles, CA; 3 Jonsson Comprehensive Cancer Center, UCLA, Los Angeles, CA; 4 UCLA AIDS Institute, UCLA, Los Angeles, CA; 5 Department of Medicine and Comprehensive Cancer Center, Cedars-Sinai Medical Center, West Hollywood, CA; 6 Department of Medicine, Division of Hematology/Oncology, AIDS Institute, UCLA, Los Angeles, California; 7 Department of Human Genetics, UCLA, Los Angeles, California; 8 Biomathematics, David Geffen School of Medicine, UCLA, Los Angeles, California; 9 Department of Biostatistics, School of Public Health, UCLA, Los Angeles, California; 10 Department of Epidemiology, Johns Hopkins Bloomberg School of Public Health, Baltimore, MD; 11 University of Pittsburgh Cancer Institute, Pittsburgh, PA; 12 Department of Medicine, Northwestern University Feinberg School of Medicine, Chicago Illinois.

**Keywords:** AIDS, Kaposi’s sarcoma, inflammatory biomarkers

## Abstract

AIDS-related Kaposi’s sarcoma (AIDS-KS) risk remains substantially elevated compared with the general population, even among patients who receive effective combination antiretroviral therapy. This study investigated the role of inflammatory and immune activating biomarkers in AIDS-KS in men who have sex with men in the Multicenter AIDS Cohort study between 1984 and 2010. Concentrations of 24 serum biomarkers; IL-1β, IL-2, IL-6, IL-8, IL-10, IL-12p70, sGP130, sIL-2Rα, sIL-6R, eotaxin, MCP-1, MCP4, MIP 1β, TARC, BLC-BCA1, IP-10, GM-CSF, IFN-γ, BAFF, sCD14, CD27, sTNFR-2, sCRP, and TNF-α were tested longitudinally in 1,501 men. The concentrations of each biomarker were compared between AIDS-KS cases and controls at multiple time points, 0–1 years, 1–2 years, 2–3 year, 3–5 years and over 5 years, prior to KS diagnosis or study termination, using univariate non-parametric Kruskal-Wallis tests and logistic regression, adjusted for HBV and HCV co-infection, race/ethnicity, age at last visit, education, smoking and CD4+ cell count. In univariate analyses, concentrations of four markers were consistently higher in cases; sIL-2Rα, IP-10, sTNFR-2, MCP-1, and five were higher in controls; GM-CSF, IL-6, MIP-1β, sCRP, sGP130. In the adjusted models concentrations of four markers were significantly inversely associated with AIDS-KS risk including sGP130 (OR=0.14, 95% CI = 0.03–0.73, BAFF (OR=0.60, 95% CI =0.16–0.90), sCRP (OR=0.61, 95% CI = 0.43–0.87) and IL-6 (OR=0.51, 95% CI = 0.35–0.76). These results support a role for markers of immune activation and inflammation in AIDS-KS and may highlight pathways to be targeted for risk stratification or therapeutics.

## Introduction:

AIDS related Kaposi’s sarcoma (AIDS-KS) was one of the first HIV related complications categorized as an AIDS defining illness. This cancer was studied extensively at the beginning of the epidemic, as there is an approximate 30% risk of development if co-infected with HIV and the human herpes virus 8 (HHV-8) and untreated over 10 years (1, 2). After highly active antiretroviral therapy (HAART) was introduced, it was expected the rates of AIDS-KS would decrease back to pre-epidemic levels in the US, approximately 3 cases per 1000 person-years from the height of epidemic at 25 cases per 1000 person-years (2). However, these rates only subsided to approximately 7.5 cases per 1000 person-years, adding significant morbidity and mortality for those who are HIV positive (2, 3, 4, 5, 6).

HHV-8 was discovered in 1994, well after AIDS-KS had been an active concern in the HIV epidemic (7, 8). The virus is necessary for AIDS-KS to occur and is estimated to have a 20–77% prevalence in high-risk HIV positive populations (1, 9, 10, 11, 12). HHV-8 is transmitted horizontally and vertically via blood, transplant-related transmission or sexual contact and is generally asymptomatic in healthy populations_13_. HHV-8 is a lifelong infection for which there are currently no treatments available (12).

HHV-8 targets the lymphatic system where, after a brief lytic phase, usually enters a dormant latent phase, which causes low levels of inflammation (10, 12, 14, 15, 16). In those with a suppressed immune system, HHV-8 can cycle back into the lytic phase, causing chronic inflammation and activated immune response (10, 12, 17, 18, 19, 20, 21, 22). This dual inflammation from HHV-8 and HIV can fatigue the immune system by introducing additional target cells for both viruses and diminishing the resources to fight these and other infections over time (10, 12, 16, 17, 18, 19, 20, 21, 22).

This inflammation can be measured via cytokines and chemokines using peripheral blood, which provides the opportunity to investigate whether there is a biological pattern of inflammation and immune response prior to AIDS-KS development. This traceable biological response to HHV-8 and HIV co-infection and the subsequent development of AIDS-KS may allow an opportunity to develop a blood based screening test for an at risk population to help with early diagnosis and minimize AIDS-KS related morbidity and mortality.

The goal of this study was to assess the concentrations of inflammatory and immune activation biomarkers at specific time points, 0–1 years, 1–2 years, 2–3 years, 3–5 years and over 5 years prior to diagnosis and, by use of step wise logistic regression, to determine biomarkers that may be predictive of AIDS-KS development at 2–3 years prior to diagnosis. This was done with a nested case control study of men who have sex with men.

## Methods:

### Population Description:

This protocol has been approved by University of California- Los Angeles Institutional Review Board 1: 10–001677. Participants were selected from the Multicenter AIDS Cohort Study (MACS), which is a prospective cohort of men who have sex with men that began in 1988 and has had three major recruiting periods; 1987–1991, 2001–2003 and 2014-present. Four academic institutions in Baltimore, Chicago, Los Angeles and Pittsburgh recruited a total of 6,972 men. These men participate in in-person interviews, computer assisted interviews, as well as physical exams, blood collection and clinical lab work at biannual visits. Information regarding the HIV’s natural history, psychological factors, medical history, demographics, treatment effects and other clinical manifestations of HIV are assessed at these visits. The MACS has been comprehensively described elsewhere (23, 24, 25).

In 2009 the MACS began the ARRA1 biomarker sub-study with the goal of using the cohort’s repository of blood samples to assess the effects of immune activation and inflammation in the progression of HIV infection. ARRA1 focused on those participants in the MACS who were long term non-progressors, those who advanced to AIDS defining illnesses (including cancer) and those who were HAART initiators. There were a total of 1885 subjects whose samples were selected and evaluated for biomarkers of immune activation and inflammation. This study is focused on the 24 immune activation and inflammation biomarkers selected by the MACS and were known the be associated with immune activation and/or systemic inflammation (26, 27). The MACS and all resulting studies using this data have been reviewed and approved annually by the Institutional Review Board for each participating institution.

### Data Acquisition and Quality Control

Data sets including a complete summary of all ARRA1 biomarkers, participants’ longitudinal biomarker data, and time varying confounding information were provided by the MACS consortium. Confounding variables included smoking, alcohol use, injection drug use, sexual activity, body mass index, co-infections, HIV seroconversion dates, first and last dates of HIV treatment, comorbidities, race/ethnicity, date of birth, and education. Cancer diagnosis information. AIDS-KS diagnoses were captured through self-report verified by pathology reports, autopsy, or through matching with cancer registers.

The data sets were combined to create a large composite longitudinal data set. In several cases, the visits from the individual longitudinal data sets were differing, resulting in missing data. If biomarkers and covariate data were missing, the value from the previous visit was used. Variables for time prior to diagnosis for cases or the end of the study timeframe for controls in days was created based on the last visit date or diagnosis date provided by the MACS consortium. All visits prior to HIV seropositivity and after AIDS-KS diagnosis were excluded.

### Covariates and confounding variables

The potential confounders and covariates were assessed for missing values, outliers and skewed distributions. The averages and standard errors (SE) for potential covariates for the time period used in the logistic regressions can be found in Table 1 for all participants and stratified by case status. Correlations between the potential covariates was also assessed and no correlation was greater than 0.75.

### Biomarker variables

Twenty-four inflammation and immune activation biomarkers were assessed including: Interleukins: (IL) 1β, 2, 6, 8, 10, 12p70 and glycoprotein 130 (sGP130) as well as receptors , sIL-2Rα and sIL-6R; chemokines: eotaxin, MCP-1, MCP4, MIP 1β , TARC, BLC-BCA1 and IP-10; and other markers including: GM-CSF, IFN-γ , BAFF, sCD14, CD27, sTNFR-2, sCRP, and TNF-α. All of these markers except for sCRP were measured using multiplex immunometric assays, using two multiplex assay platforms (MesoScale Discovery-MSD or Luminex). sCRP was measured by Quest Diagnostics using a clinically validated assay. All samples were run at the same time for each participant (26, 27).

Each of the biomarkers were assessed for outliers, missing values and skewness. Most markers were skewed to the left with extreme outliers. For biomarkers with values below the detectable level, they were revalued as half of the lowest limit for the biomarker and values above the detectable level assigned the highest value. There were five biomarkers that had greater than 10% of values were that were below the lowest detectable limit; IL-1β, GM-CSF and IFN-γ were missing in approximately 30% of study participants, IL-2 was missing 18% and IL-12p70 was missing for 11% of the study population. IL-10 and IL-12p70 were statistically significantly correlated (r=0.77), as well asIL-1β and IL-6 (r=0.80).

### Analysis strategies

**Figure 1:**
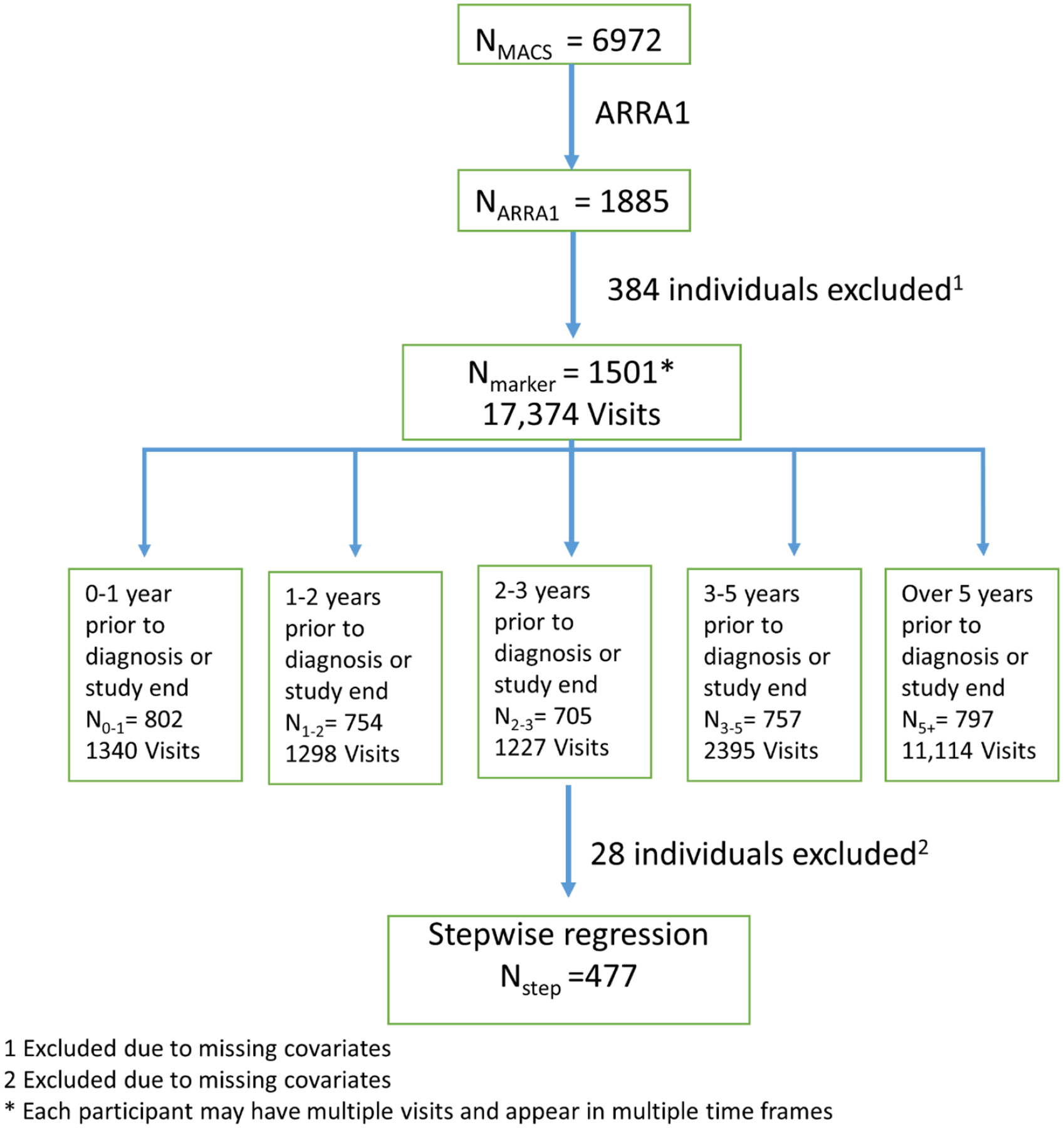
Study Design Schematic of the Available Number of Subjects

The average concentration of each biomarker was estimated for all participants, for AIDS-KS cases only and for unaffecteds only. Visits were then divided into five time points, 0–1 years prior to diagnosis, 1–2 years prior, 2–3 years prior, 3–5 years prior and greater than 5 years prior to diagnosis. The average concentrations for AIDS-KS cases and non-cases was found for each time window. Non-parametric Kruskal-Wallis tests were performed to assess the statistical difference between cases and controls at each time-point. Although participants’ visits were every 6 months in the MACS, the number of case and control visits varied based on the actual visits available in that timeframe that were analyzed by the ARRA1 sub-study. The number of participants and participant visits available for each time frame are given below and in figure 1.

Analysis of the latest visit in the 2–3 year window prior to diagnosis or the participant’s last visit was used for descriptive statistics and logistic regression models. This timeframe was selected because there were the highest number of participants’ visits available and it was a notable pivot point for several biomarkers as participants approached AIDS-KS diagnosis. Each participant who was seen during this timeframe contributed 1 visit to the analysis.

Descriptive statistics, including age, BMI, CD4+ cell count, HIV viral load, race/ethnicity, education, smoking status, history of radiation treatment, history of hospitalizations in the last 6 months prior to the visit, hepatitis B (HBV) and C (HCV) infection status, cohort, alcohol use, injection drug use, HIV medication use and sexual activity, were calculated for all study members, as well as separately for AIDS-KS cases and controls.

Logistic regression was used to assess the odds of developing AIDS-KS given the natural logarithmic change in each biomarker. A univariate model was run for each biomarker and an adjusted model including age, CD4+ cell count, education status, race/ethnicity, HBV status, HCV status and smoking status using data from the 2–3 year window prior to diagnosis or the participant’s last visit. CD4+ cell count was used as a proxy for HIV medication use to correct for possible noncompliance and variability in the effectiveness of treatment over time.

All log transformed markers and the covariates were then entered into forward and backward stepwise logistic regressions in order to determine a possible predictive model for estimating risk of AIDS-KS at 2–3 years prior to diagnosis. Controls were selected based on participation in the ARRA sub-study for at least one visit during the period between 2–3 years prior to study end. The visit in this timeframe closest to diagnosis was selected if more than on visit was available. A total of 37 AIDS-KS cases and 668 controls had visits available for analysis in the 2–3 year prior to diagnosis window and were used for this part of the analysis. All statistical analyses were performed using SAS Software 9.4 (SAS Institute, Cary, NC, USA, 2018).

## Results:

### Population description

The average age at the last visit for all participants was 52.5 (SE = 12.45) years, with those participants who were AIDS-KS cases being an average of 34.58 (SE = 13.10) years old and controls were 53.5 (SE = 11.64) years old. The average BMI was 23.76 (SE = 3.66) kg/m^2^ in AIDS-KS cases and controls were slightly overweight with an average of 25.42 (SE = 4.39) kg/m^2^. The average CD4+ cell count and viral load varied significantly between cases and controls with cases having cell counts of 373.89 (SE = 156.08) cells and a viral load of 100,579 (SE =130,706) copies/ml, while controls had 582.04 (SE =298.36) cells and a viral load of 37,944.65 (SE =271,527) copies/ml. There was also a significant difference in the type of therapy for HIV used by cases and controls. Among cases 51.61% took no therapy compared to 11.66% of controls; 22.58% of cases had monotherapy compared to 3.37% of controls; 9.68% of cases took combination therapy compared to 6.90% of controls and only 16.13% of cases took potent ART compared to 78.07% of controls.

Subjects in both groups were more likely to be white, with 83.78% of cases and 58.53% of controls identifying as white. In addition, 8.11% of cases and 27.84% of controls identified as African American and 2.7% of cases and 11.64% of controls identified as Hispanic. The cases were more likely to have completed high school or college. 47.22% of cases earned a high school diploma and 52.78% of cases graduated college. Of the controls 8.25% did not complete high school, while 50.22% have a high school diploma as their highest educational attainment and 41.53% having finished college. Cases were more likely to be recruited at the Los Angeles site, with 48.65% of them originating there compared to 27.84% of controls, while only 5.41% of cases came from Baltimore compared to 26.65% of controls. AIDS-KS cases were also more common in the 1984 cohort with 64.86% of cases from this time compared to 35.93% of controls. The controls were most likely to be from the 2010 period, from which 41.92% of controls and 10.81% of cases originated.

The rates of HBV and HCV infection were similar in both groups, as was radiation exposure, hospital stays within 6 months prior to the visit and the use of alcohol and steroids. The use of anti-inflammatory drugs and statins were both higher in the control group compared to the case group (42.51% of controls vs. 21.62% of cases and 25.60% of controls vs. 2.75% of cases respectively). Cases were more likely to be current smokers (62.16%) compared to controls (42.22%) and were more likely to have had sexual activity since their last visit, with 94.59% of cases reporting activity compared to 66.36% of controls. A detailed description of demographic information for the population is located in Table 1.

### Analysis of Biomarker Concentration Averages

#### Over 5 Years Prior to Diagnosis

There were 11,114 visits from 797 participants that took place over five years prior to diagnosis that included 92 visits from 21 AIDS-KS cases and 11,022 visits from 776 controls. sIL-2Rα, sIL-6R, IL-10, IP-10, MCP-1, TNF-α, eotaxin, BLC-BCA1, CD27 and sTNFR-2 were all statistically significantly higher in AIDS-KS than controls. sGP130, IL-2, IL-6, sCRP, MCP-4, TARC and MIP-1β were significantly lower in AIDS-KS cases than controls.

#### 3 to 5 Years Prior to Diagnosis

Between three and five years prior to diagnosis there were 2,395 total visits from 757 participants including 96 from 33 AIDS-KS cases and 2,299 visits from 724 controls. Eleven of the biomarkers were statistically elevated in AIDS-KS cases compared to controls including: IFN-γ, sIL-2Rα, sIL-6R, IL-10, IP-10, MCP-1, TNF-α, BAFF, BLC-BCA1, CD27 and sTNFR-2. Seven markers were higher in controls than cases during these two years, including; IL-1β, IL-2, IL-6, MCP-4, MIP-1β, sGP130 and sCRP. All markers maintained their general trends from the previous time point.

#### 2 to 3 Years Prior to Diagnosis

The analysis of two to three years prior to diagnosis included 1,227 visits from 705 participants, 37 of which were AIDS-KS cases contributing 72 visits and 668 were controls contributing 1,155 visits. There were seven biomarkers that continued to be statistically significantly higher in controls than cases, IL-2, sIL-6R, IP-10, BAFF, BLC-BCA1, CD27 and sIL-2Rα, while nine were higher in controls than cases: IFN-γ, sGP130, IL-8, IL-10, TNF-α, sCRP, sTNFR-2, MCP-4 and MIP-1β.

This time point was a pivoting point for several of the biomarkers. IL-8 and sCRP were considerably lower in AIDS-KS cases than controls up to this point, but as diagnosis approached, levels in cases made dramatic shifts to equalizing and exceeding the concentrations found in controls. The other biomarkers generally maintained their trends, with the exception of IL-10 and IL-12p70, in which cases spiked lower and higher, respectively, than controls, but the differences were not statistically significant for IL-12p70.

#### 1 to 2 Years Prior to Diagnosis

The analysis for the two years prior to diagnosis was conducted by only including visits that occurred in between the one year mark and two year mark prior to diagnosis. There were 1,298 visits from 754 participants with 58 visits of 35 AIDS-KS cases and 1,240 visits from 719 controls, which were all available visits from the ARRA1 sub-study during this timeframe. The averages of each biomarker were found within the 2-year time restriction. Sixteen of the markers were found to be statistically significantly different in the AIDS-KS case visits compared to the unaffected visits. Eotaxin, IL-2, sGP130, BAFF, CD27 and MIP-1β were all statistically significantly higher in the controls, than in the subsequently affecteds. IFN-γ, sIL-2Rα, sIL-6R, IL-10, IP-10, TNF-α, MCP-1, sCD14, BLC-BCA1 and sTNFR-2 were found to be higher in the subsequent AIDS-KS cases than those unaffected. All other biomarkers maintained their general trend, but no longer met statistical significance, with the exceptions of sCRP and IL-8 for which the group with the higher level changed, but these relationships were not statistically significant.

#### 0 to 1 Year Prior to Diagnosis

The analysis for one year prior to diagnosis was conducted using only visits that occurred in the year prior to an AIDS-KS diagnosis or the participant’s last visit. There were a total of 1,340 visits from 802 participants, 89 AIDS-KS case visits from 39 participants and 1,251 control visits from 763 participants. The means of each biomarker was found within the time restrictions and a Kruskal-Wallis test was conducted to determine the statistical significance. Fifteen of the 24 markers were found to be statistically significantly different in the AIDS-KS case visits compared to the unaffected visits. IL-1β, IL-2, MIP 1β and sGP130 were all statistically significantly higher in the controls, than in the subsequently affecteds. IFN-γ, sIL-2Rα, sIL-6R, IL-10, BAFF, sCD14, sIL-2Rα, sTNFR-2, IP-10, MCP-1, CD27 and TNF-α were found to be higher in the subsequent AIDS-KS cases than in the controls.

#### All Available Visits Prior to Diagnosis

There were a total of 17,374 visits analyzed with 407 visits from 79 individuals who developed AIDS-KS and 16,967 visits from 1422 individuals who did not. The means of each of the biomarkers were determined and then the means stratified by case status. Twenty of the 24 biomarkers’ means were statistically significant different over the course of all study visits. Those with statistically significantly higher values in the AIDS-KS cases were IFN-γ, MCP-1, IFN-γ, sIL-6R, sIL-2Rα, IL-10, BLC-BCA1, CD27, sTNFR-2, sCD14, TNF-α, and IP-10. Those with statistically significantly higher in the those unaffected were, IL-1β, IL-2, BAFF, sGP130, IL-8, IL-6, sCRP, MCP-4, MIP 1β and TARC.

### Logistic Regression Analysis

The logistic regression analysis was performed using data from the visit closest to diagnosis between 2 and 3 years prior to diagnosis. There were 705 available participants in this timeframe, each contributing one visit, including 37 AIDS-KS cases and 668 controls. Each biomarker was log transformed and entered into a univariate logistic regression model and a logistic model adjusted for HBV co-infection, HCV co-infection, race/ethnicity, age at last visit, education, smoking and CD4+ cell count.

In the univariate models, six markers showed an increased risk of AIDS-KS when these were elevated. These biomarkers include IFN-γ (OR= 1.19; CI= .02, 1.38; p=.0257), IL-1β (OR=1.18; CI= .00, 1.39; p=.0484), IL-2 (OR=1.18; CI=.01, 1.39; p=.0438), sIL-2Rα (OR=2.86; CI=.64, 4.95, p=.0002), IL-10 (OR=1.46; CI=.19, 2.54; p=.001) and IP-10 (OR=1.74; CI=.19, 2.54 p=.0043). Two markers that had lower values showed an increased risk of AIDS-KS, including MIP-1β (OR=.62; CI=.39, .98; p=.0400) and sGP130 (OR=.22; CI=.05, .93; p=.0399).

**Figure 2:**
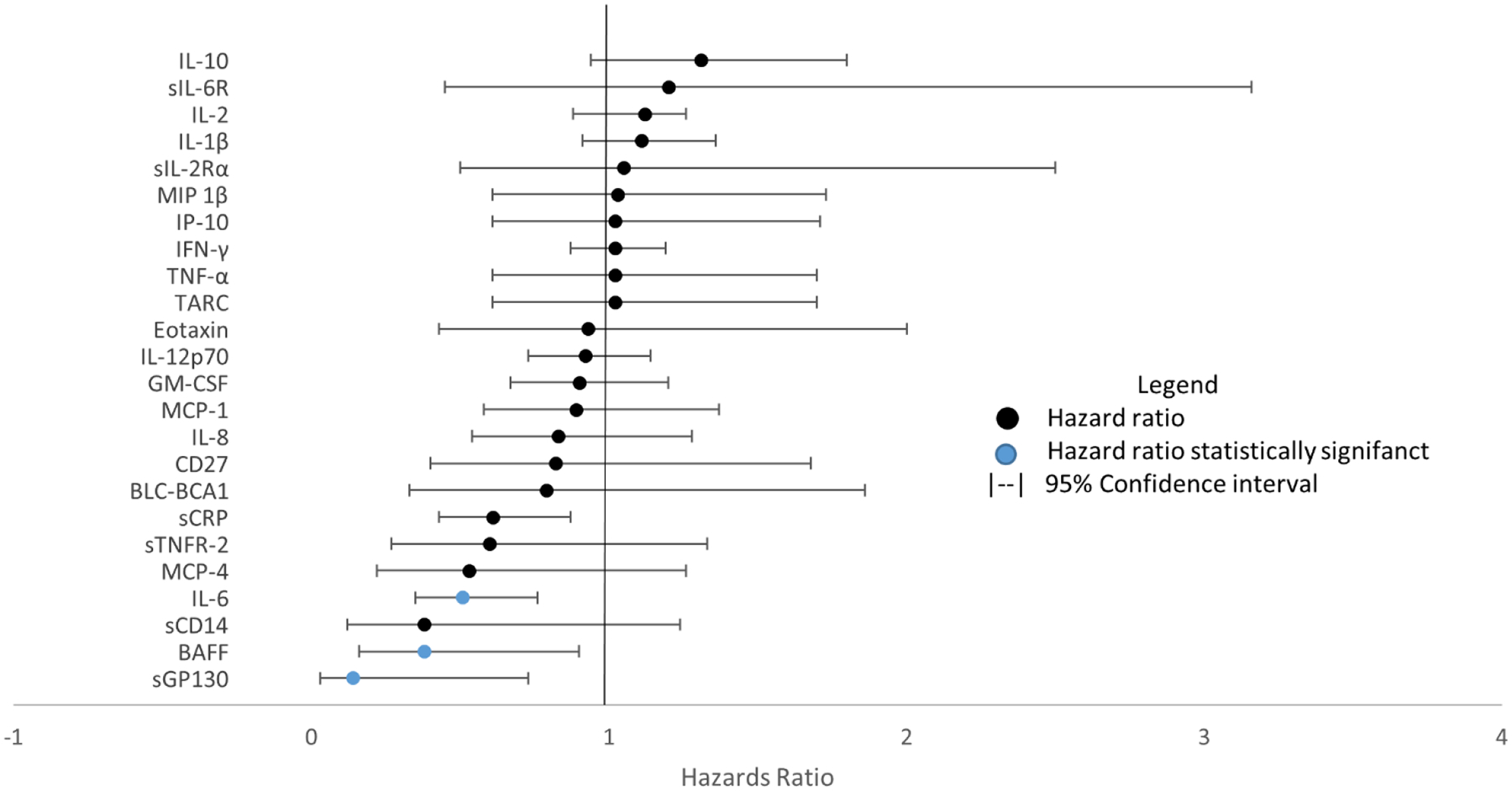
Odds ratios of transformed markers in models adjusted for HBV coinfection, HCV coinfection, age at last visit, race/ethnicity, education, smoking and CD4+ cell count with 95% Wald confidence intervals. Black markers represent non-statistically significant ratios and blue indicates statistical significance.

In the adjusted models there were no markers that were statistically significantly associated with an increased risk in AIDS-KS. There were four markers that inversely associated with AIDS-KS sGP130 (OR=0.14; CI= 0.03, 0.73; p=.0197), BAFF (OR=.60; CI=.16, .90p=.0282), sCRP (OR=.61; CI=.43, .87; p=.0064) and IL-6 (OR=.51; CI= .35, .76; p=.0009) (Figure 2). The adjusted models were statistically significantly better fitting models as assessed by the likelihood ratio test.

### Step-Wise Logistic Regression

All biomarkers and covariates from the adjusted logistic model were used for forward and backward stepwise logistic regression models. At each step in the backwards selection the criterion to remove a variable was that it be the least significant variable whose p-value was greater than 0.15. Likewise, at each step the criterion for allowing a variable to enter the regression model was that it be the most significant variable whose p-value was less than .10. Fisher’s scoring was used to determine the p-values. Of the 705 visits available in the 2–3 year prior to diagnosis window, 477 were used, including 25 AIDS-KS cases and 452 controls, due to incomplete data for covariates.

**Figure 3:**
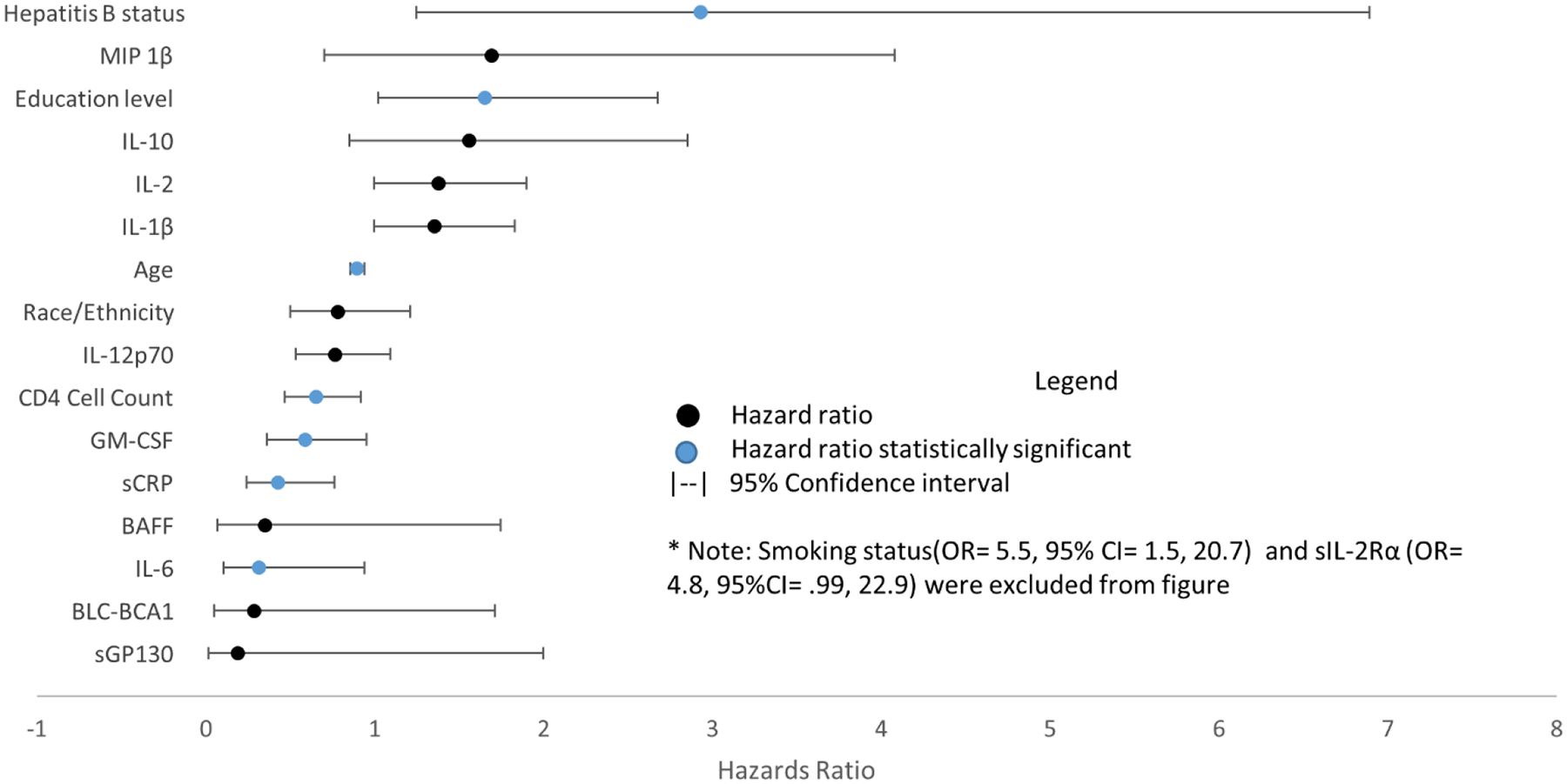
Odds ratios of transformed markers in forward stepwise regression with 95% Wald confidence intervals. Black markers represent non-statistically significant ratios and blue indicates statistical significance. Smoking status (OR=5.5, 95% CI=1.5, 20.7) and sIL-2Rα (OR=4.8, 95% CI= .99, 22.9) were excluded from the figure.

In the forward stepwise regression ten variables were found to be significant at α=.05, including age (OR= .91; 95% CI= .88, .95; p=<.0001), HBV (OR= 3.02; CI= 1.38, 6.64; p=.0059), sCRP (OR= .48; 95% CI= .28, .82; p=.0070), CD4+ cell count (OR= .69; 95% CI= .52, .91; p=.0085), sIL-2Rα (OR= 4.74; 95% CI= 1.40, 15.98; p=.0122), sGP130 (OR= .07; 95% CI= .01, .57; p=.0126), education status (OR= 1.65; CI= 1.02, 2.68; p=.0135), IL-6 (OR= .40; 95% CI= .19, .84; p=.0157), smoking status (OR= 4.30; 95% CI= 1.30, 14.23; p=.0168), and IL-1β (OR= 1.40; 95% CI= 1.06, 1.85; p=.0176) (Figure 3). Only one marker, GM-CSF (OR= .67; 95% CI= .44, 1.01; p=.0568), had a p-value between .05 and .10. The forward stepwise regression model has a Hosmer-Lemeshow goodness of fit statistic corresponding to p=.8821.

**Figure 4:**
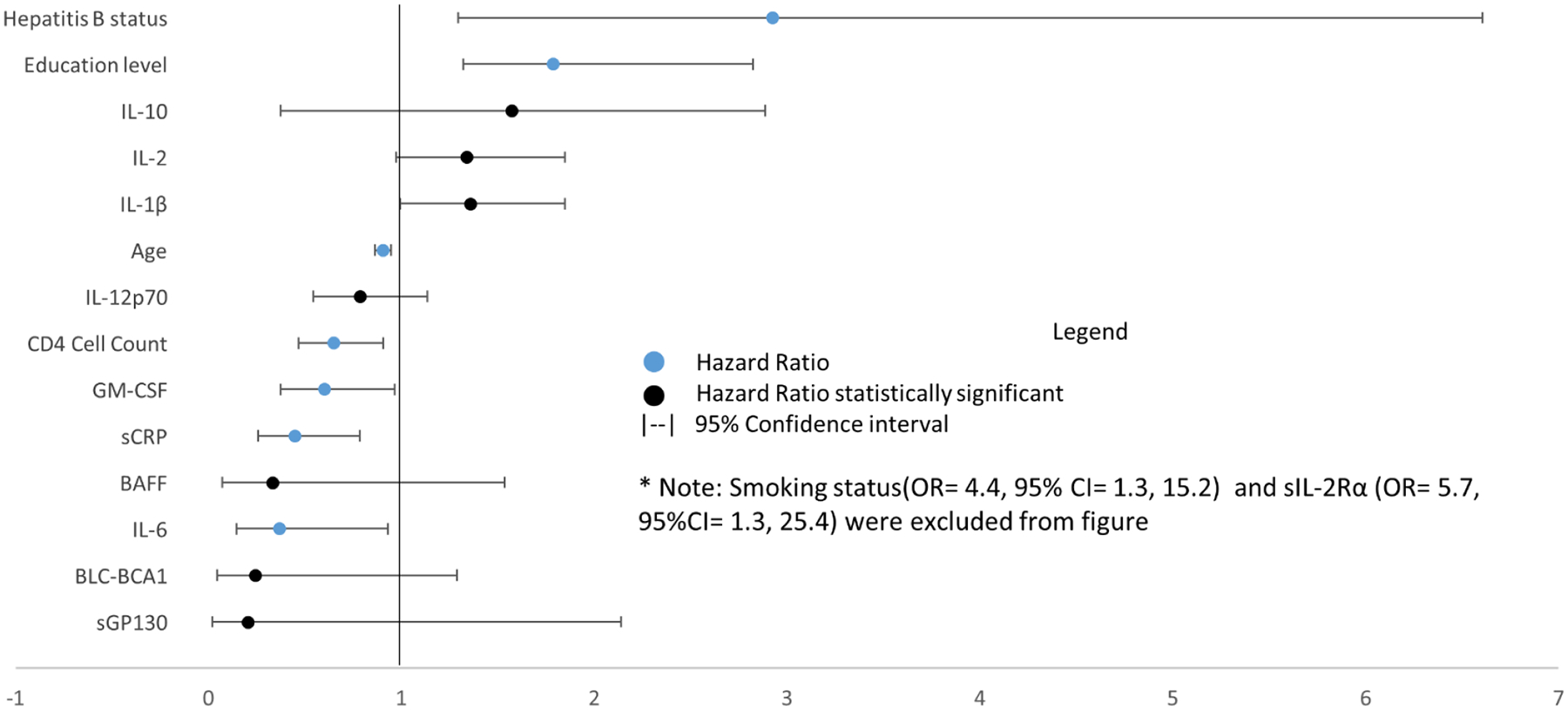
Odds ratios of transformed markers in backward stepwise regression with 95% Wald confidence intervals. Black markers represent non-statistically significant ratios and blue indicates statistical significance. Smoking status (OR=4.4, 95% CI=1.3, 15.2) and sIL-2Rα (OR=5.7, 95% CI= 1.3, 25.4) were excluded from the figure.

In the backward stepwise regression GM-CSF, IL-1β, sIL-2Rα, sCRP, age, CD4+ cell count, smoking status, HBV infection, and education status all remained in the model with similar values as the forward stepwise regression. sGP130 and IL-6, which were included in the forward model, were both eliminated with p=.2738 and p= .3231 respectively (Figure 4). In addition, in the backward model BAFF achieved a statistical significance of under a=.05 with p=.0330, which it had not in the forward model. The remaining variables all achieved similar levels of statistical significance as they had in the forward model.

## Discussion:

This study approached the comparisons of the concentrations of inflammatory and immune activating biomarkers by reviewing the average concentrations in cases and controls during multiple time points prior to diagnosis or the end of the study and by conducting a case-control analysis that allowed the inclusion of the maximum number of cases, which occurred by using 2–3 years prior to diagnosis timeframe.

The comparison of averages of the biomarkers’ concentrations did show that the groups had significantly different levels at most time points for many markers. In the 1-year prior analysis, which had the least amount of data points available, nearly half of the markers showed difference between cases and the controls. These differences in averages may inform which markers should be examined together and for those markers with differences, further analysis of the levels at earlier time points can be examined separately from an “all visit” analysis to determine when the discrepancy begins.

The observation of the changes in the averages of the biomarkers over time demonstrated not only a change in average values, but how those averages behaved over time. Many markers mirrored general trends in both groups, indicating that the changes are normal and not due to AIDS-KS development, however for sCD14, IL-12p70, IL-10 and IL-8, the trends were very different from the trends in the control group.

The analysis of the several years prior to diagnosis or the end of the study demonstrates that the biomarkers in individuals unaffected by AIDS-KS remain very stable. In most of the biomarkers, the trajectory for the cases was also stable, although different from those unaffected. The exceptions noted were IL-8, IL-10 and IL-12p70. Among these, IL-10 was seen to be increasing closer to diagnosis in those with AIDS-KS and IL-12p70 was decreasing, both of which would be consistent with reduced T_H_ cell differentiation (28). IL-8 was also seen to increase over time which may be related to their angiogenic activities; a hallmark of KS (28).

The results of the logistic regression demonstrate a significant decrease in IL-6, BAFF, sGP130 and sCRP. IL-6 was of particular interest as it was consistently lower in cases than controls. HHV-8 produces a viral version of IL-6 (vIL-6), which can independently act as IL-6 in order to increase the acute immune response, increase inflammation and promote the lytic phase of the HHV-8 lifecycle. This viral version activates the IL-6 receptor (sIL-6R), but would not be detected on the IL-6 biomarker analysis. If the viral version is readily available, it is possible the cases may have produced less IL-6, sensing it was already abundant. In addition to the IL-6 concentrations being lower, sIL-6R concentrations were consistently higher in cases than controls. This inverse relationship between the interleukin and its receptor may be related to this production as the virus becomes lytic and produces more of the viral version indicating more active HHV-8 infection (29, 30).

Consistently, sGP130 and sCRP production are related to IL-6 with sGP130 being a stabilizer for the IL-6 receptor complex and sCRP being produced by the liver when IL-6 concentrations are high (28). BAFF’s association with AIDS-KS switched from hazardous to lower concentrations indicating a lower risk once other factors were taken into account. BAFF is largely associated with B-cell proliferation and a lower level may be consistent with immune fatigue, which could lead to a pro-cancer environment (18, 21, 23, 31).

The full model included the participant’s age, race/ethnicity, CD4+ cell count, HBV co-infection, HCV co-infection, education and smoking status. Several of these variables may include errors as these were collected by survey, but in general the MACS surveys are well validated and consistent. CD4+ cell count and education were used as proxies for the advancement of HIV infection and socioeconomic status respectively and may not be perfect proxies in all cases.

The ARRA1 sub-study, from which these data originated, may also influence the results as the original study population selected was not specifically for the study of AIDS-KS. The participants may have developed AIDS-KS after the study period, which if within a close proximity to the end of the study, may have biased the results toward the null. In addition those selected for ARRA1 may have had other AIDS defining illnesses, be a long term non-progressor or have other special circumstances that made them desirable to include. This may create a control population that is not a true reflection of those at risk for AIDS-KS outside of the study.

This study was able to utilize all available cases of AIDS-KS in the ARRA1 sub-study of the MACS, which is one of the largest available datasets to investigate the effect of inflammation and immune activation prior to AIDS-KS diagnosis. Decreased levels of IL-6 and its downstream targets, sGP130 and sCRP, as well as BAFF, correlated with a greater risk of AIDS-KS. This information can inform future research to investigate a possible model for early detection of AIDS-KS in at risk men.

## Figures and Tables

**Figure 5.**
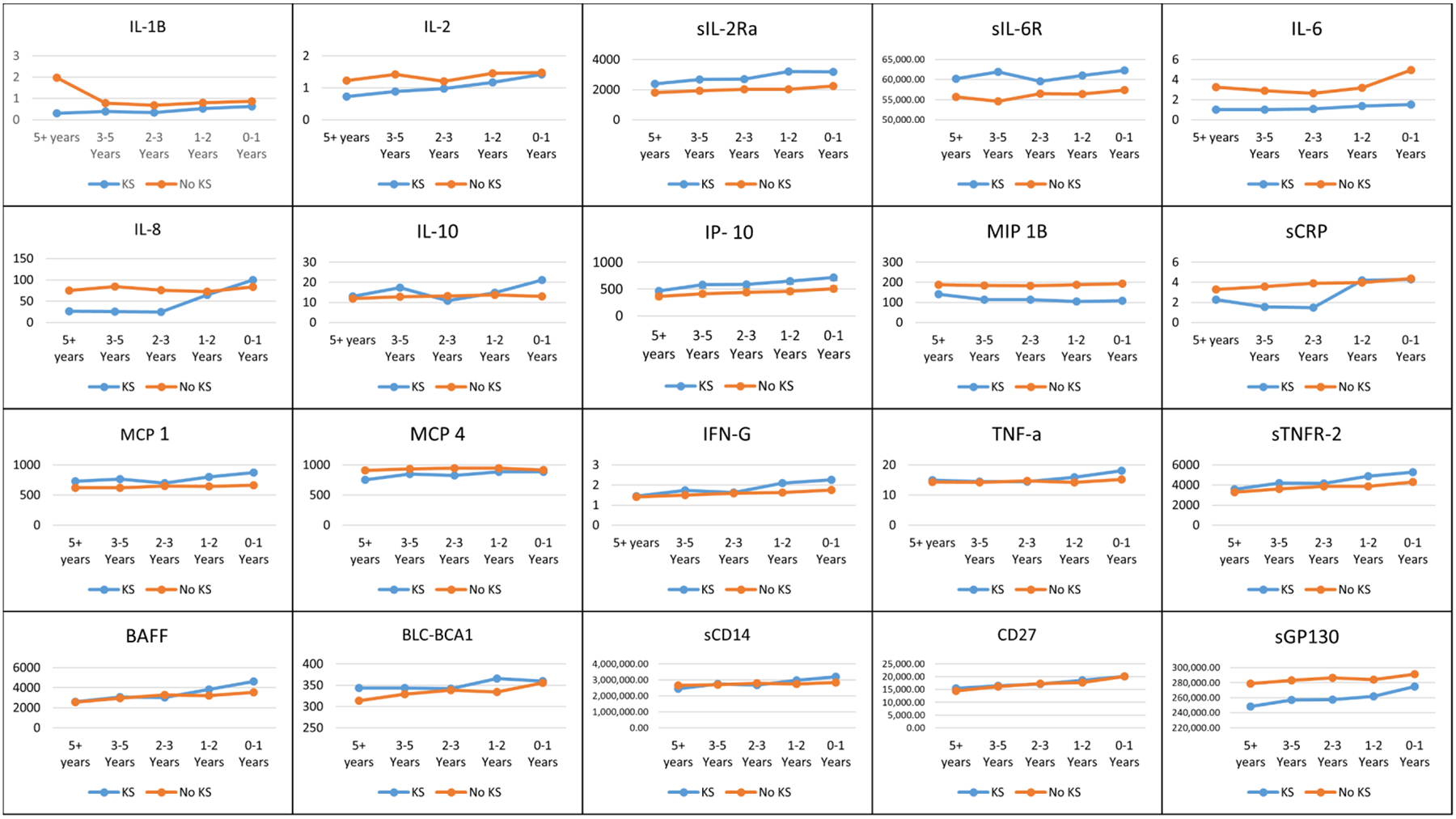
Average biomarker levels for AIDS-KS cases and unaffecteds prior to diagnosis or study termination.

**Table 1:** Demographics at last visit between 2–3 years prior to diagnosis

	Total (%)	KS (%)	No KS (%)	P-Value^t^
Average age (years)*	52.49 (+/− 12.45)	34.58 (+/− 13.10)	53.49 (+/− 11.64)	**<.0001**
Average BMI*	25.33 (+/− 4.37)	23.76 (+/− 3.66)	25.42 (+/− 4.39)	**.0114**
Average CD4+ cell count*	570.75 (+/− 295.18)	373.89 (+/− 156.08)	582.04 (+/− 297.36)	**<.0001**
Average viral load*	40,503.12 (+/− 267,466.02)	100,579.85 (+/− 130,705.96)	37,944.65 (+/− 271,527.61)	**.0280**
Average time since HIV infection in years*	15.11 (+/− 9.91)	7.11 (+/− 5.03)	15.55 (+/− 5.03)	**<.0001**
**Race**	705	37	668	**.0236**
White	422 (59.86)	31 (83.78)	391 (58.53)	
African American	189 (26.81)	3 (8.11)	186 (27.84)	
Hispanic	43 (6.10)	0 (0)	43 (6.10)	
Hispanic/African American	3 (.43)	0 (0)	3 (.45)	
Asian or Pacific Islander	2 (.28)	1 (2.70)	1 (.15)	
American Indian or Alaskan Native	2 (.28)	0 (0)	2 (.30)	
Other	6 (.85)	1 (2.70)	5 (.75)	
Other Hispanic	38 (5.39)	1 (2.70)	37 (5.54)	
**Education**	703	36	667	**.0138**
Less than 9^th^ Grade	14 (1.99)	0 (0)	14 (2.10)	
9–11^th^ Grade	41 (5.83)	0 (0)	41 (6.15)	
12^th^ Grade	127 (18.07)	3 (8.33)	124 (18.59)	
College- Less than 4 years	255 (32.01)	14 (38.89)	211 (31.63)	
College- 4 years	135 (19.20)	10 (27.78)	125 (18.74)	
Some Graduate	52 (7.40)	7 (19.44)	45 (6.75)	
Post Graduate	109 (15.50)	2 (5.56)	107 (16.04)	
Smoker*	686	37	649	**.0254**
Current	297 (43.29)	23 (62.16)	274 (42.22)	
Former	389 (56.71)	14 (37.84)	375 (57.78)	
**Site**	705	37	668	**.0085**
Baltimore	180 (25.53)	2 (5.41)	178 (26.65)	
Chicago	182 (25.82)	9 (24.32)	173 (25.90)	
Pittsburgh	139 (19.72)	8 (21.62)	131 (19.61)	
Los Angeles	204 (28.94)	18 (48.65)	186 (27.84)	
**Cohort**	705	37	668	**.0004**
1984	264 (37.45)	24 (64.86)	240 (35.93)	
1987	94 (13.33)	8 (21.62)	86 (12.87)	
2001–2003	63 (8.94)	1 (2.70)	62 (9.28)	
2010+	284 (40.28)	4 (10.81)	280 (41.92)	
HIV therapy type*	683	31	652	**<.0001**
No therapy	92 (13.47)	16 (51.61)	76 (11.66)	
Monotherapy	29 (4.25)	7 (22.58)	22 (3.37)	
Combination therapy	48 (7.03)	3 (9.68)	45 (6.90)	
Potent ART	514 (75.26)	5 (16.13)	509 (78.07)	
Alcohol intake*	686	37	649	.0859
None	155 (22.59)	4 (10.81)	151 (23.27)	
1–3 drinks/week	358 (52.19)	18 (48.65)	340 (52.39)	
4–13 drinks/week	113 (16.47)	9 (24.32)	104 (16.02)	
13+ drinks/week	60 (8.75)	6 (16.22)	54 (8.32)	

* At last visit before censoring

t Pearson’s Chi Squared Test Exact

## Abbreviations:

AIDS: Acquired Immune Deficiency Syndrome
AIDS-KS: Acquired Immune Deficiency Syndrome Related Kaposi’s Sarcoma
BAFF: B-Cell Activating Factor
BLC/BCA1: B Lymphocyte Chemoattractant/ B Cell Attracting Chemokine-1
BMI: Body Mass Index
CD4: Cell Differentiation 4 Positive
GM-CSF: Granulocyte Macrophage Colony Stimulating Factor
HAART: Highly Active Anti-Retroviral Therapy
HHV-8: Human Herpes Virus 8
IFN-γ: Interferon- Gamma
IL-1β: Interleukin 1-Beta
IL-2: Interleukin 2
IL-6: Interleukin 6
IL-8: Interleukin 8
IL-10: Interleukin 10
IL-12: Interleukin 12
IP-10: Interferon Gamma Producing Protein 10
KS: Kaposi’s Sarcoma
MCP1: Monocyte Chemoattractant Protein 1
MCP4: Monocyte Chemoattractant Protein 4
MIP1β: Macrophage Inflammatory Protein 1-Beta
sCD14: Soluble Cluster Differentiation 14
sCRP: Soluble C Reactive Protein
sGP130: Soluble Glycoprotein 130
sIL2Rα: Soluble Interleukin 2 Receptor Alpha
sIL6R: Soluble Interleukin 6 Receptor
sTNFR2: Soluble Tumor Necrosis Factor Receptor 2
TARC: Thymus and Activation Regulator Chemokine
TNF-α: Tumor Necrosis Factor Alpha
vIL-6: Viral Interleukin 6
